# Early Surgery or Conservative Management for Adhesive Small Bowel Obstruction: A Comprehensive Systematic Review of Short- and Long-Term Outcomes

**DOI:** 10.7759/cureus.101184

**Published:** 2026-01-09

**Authors:** Fahd Al Abbood, Omar Altamimi, Ibrahim Altamimi, AlJouhrah M AlAbdullah, Fatimah A Alkhars, Abdulrahim Bamagos, Rawiyah A Alkabkabi, Rahaf A Alharthi, Abdulkreem Al-Juhani

**Affiliations:** 1 General Surgery, Saudi Board of General Surgery, King Salman Hospital, Riyadh, SAU; 2 Surgery, Faculty of Medicine, King Abdulaziz University Hospital, Jeddah, SAU; 3 Surgery, Diriyah Hospital, Riyadh, SAU; 4 Medicine and Surgery, Almaarefa University, Riyadh, SAU; 5 General Practice, Almaarefa University, Riyadh, SAU; 6 College of Medicine, King Saud Bin Abdulaziz University for Health Sciences, Makkah, SAU; 7 College of Medicine, Umm Al-Qura University, Makkah, SAU; 8 Forensic Medicine, Forensic Medicine Center, Jeddah, SAU; 9 Surgery, Faculty of Medicine, King Abdulaziz University, Jeddah, SAU

**Keywords:** adhesive small bowel obstruction, bowel ischemia, early surgery, gastrografin, non-operative management, paediatric sbo, recurrence

## Abstract

Adhesive small bowel obstruction (aSBO) is a common cause of emergency surgical admission, and the optimal initial management between early surgery and conservative non-operative management (NOM) remains debated, particularly regarding recurrence and short-term safety. This systematic review aimed to evaluate short- and long-term outcomes of early operative management compared with conservative treatment for aSBO in adult and paediatric populations. A systematic search of MEDLINE, Embase, Scopus, and Cochrane CENTRAL was conducted from inception to 2025 in accordance with PRISMA guidelines. Nine studies were included. In stable adults, NOM was generally safe with comparable short-term complication and mortality rates to early surgery; however, prolonged delays following NOM failure were associated with increased morbidity and bowel resection. Long-term outcomes consistently favoured early operative management, demonstrating a 40-60% relative reduction in recurrence. Water-soluble contrast protocols improved diagnostic accuracy and NOM success, while specific radiological findings predicted failure. In paediatric patients, NOM was effective in many cases, but infants under one year of age had a higher likelihood of requiring surgery, and delays beyond 48 hours increased bowel resection rates. Overall, NOM is safe for selected patients, but early surgery offers superior long-term recurrence outcomes.

## Introduction and background

Adhesive small bowel obstruction (aSBO) is still one of the leading reasons of emergency surgical admission globally, accounting for up to 75% of all small intestine obstructions and placing a significant clinical and economic burden on modern health systems [1]. The majority of instances result from postoperative intra-abdominal adhesions, which form in up to 93% of individuals following major abdominal surgery and can cause recurrent obstructive occurrences years later [2]. Although conservative management, typically combining bowel rest, nasogastric decompression, and fluid resuscitation, has traditionally been considered the first line of treatment for stable patients, the best balance between early surgery and non-operative management (NOM) is still being contested [3]. 

Recognising the danger of development to ischemia or strangulation, which can be modest but is linked with significant morbidity when surgical intervention is delayed, is an important aspect of modern decision making [4]. At the same time, surgery itself has dangers such as intestinal damage, postoperative difficulties, and the possibility of future adhesion formation. Long-term outcomes also vary significantly: While a single surgical episode may lessen the probability of future recurrence, each subsequent recurrence might have a negative impact on quality of life and increase healthcare utilisation [5-9]. 

The dilemma of whether early operational intervention or initial conservative care leads to superior short- and long-term outcomes remains therapeutically important for both adult and pediatric populations. Despite various cohort studies addressing facets of this challenge, the evidence has not been comprehensively synthesised to guide practice in a variety of health-care settings and ages. 

This systematic review intends to compare the outcomes of early surgery against conservative care in a SBO, with an emphasis on short-term morbidity, mortality, bowel resection, and long-term recurrence.

## Review

Methodology 

*Study Protocol* 

**Figure 1:**
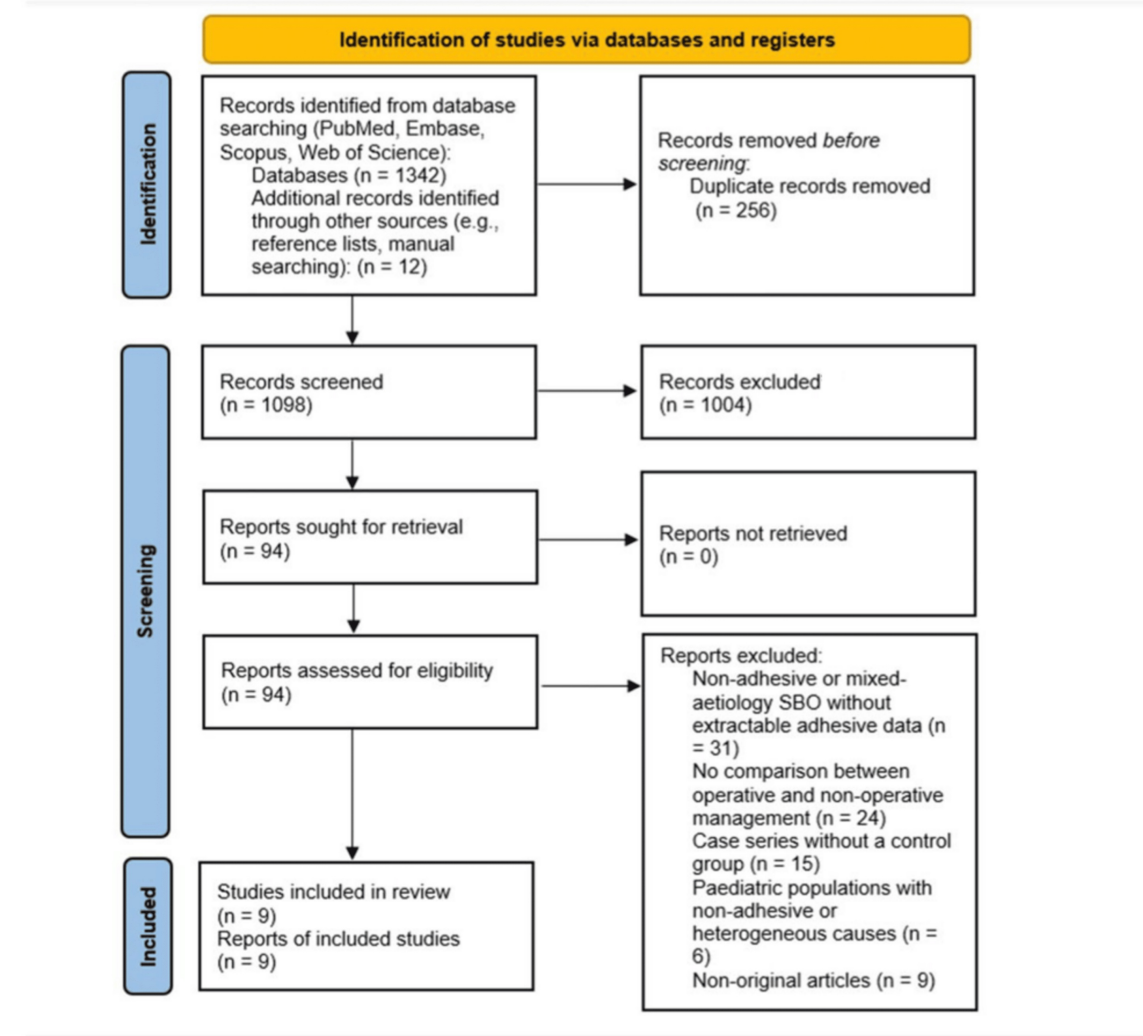
PRISMA flow diagram. PRISMA: Preferred Reporting Items for Systematic reviews and Meta-Analyses.

This systematic review was conducted in accordance with Preferred Reporting Items for Systematic reviews and Meta-Analyses (PRISMA) 2020 guidelines (Figure 1) [10]. The protocol was developed a priori and included predefined eligibility criteria, outcomes, and analysis methods.

*Eligibility Criteria* 

We included peer-reviewed primary studies evaluating operative management versus NOM of aSBO in adult or pediatric populations. Eligible study designs were randomised trials, prospective or retrospective cohort studies, and case-control studies published in English from database inception to 2025.

Studies were included if they reported outcomes for aSBO (postoperative or post-inflammatory), and/or compared early or index operative management with conservative management/NOM, or evaluated outcomes related to the timing of surgery after a trial of NOM.

We excluded studies if they included mixed causes of SBO without extractable adhesive data, lacked a comparison group, were case series, reviews, editorials, or conference abstracts, and reported purely early postoperative ileus or malignant/hernia-related SBO.

Information Sources and Search Strategies

Systematic searches were conducted in MEDLINE (PubMed), Embase, Scopus, and Cochrane CENTRAL. Reference lists of included articles and related reviews were screened manually. Search strategies combined controlled vocabulary and keywords related to small bowel obstruction, adhesions, operative management, NOM, and timing of surgery. The full search strategy is available upon request.

Study Selection

Titles and abstracts were screened independently by two reviewers. Full texts of potentially relevant articles were then assessed against the eligibility criteria. Discrepancies were resolved through discussion. A PRISMA flow diagram summarises the selection process. Ultimately, nine studies were included.

*Data Extraction* 

Data extraction was performed independently by two reviewers using a structured template. For each study, we collected information on design, setting, population, definitions of early surgery and NOM, and baseline clinical characteristics. We extracted all relevant outcomes, including short-term complications, bowel ischemia or resection, length of stay, mortality, and long-term recurrence. Details on predictors of NOM failure or recurrence were also recorded when reported. Extracted data were compared, and discrepancies were resolved through consensus to ensure accuracy and completeness.

*Risk of Bias Assessment* 

Risk of bias in observational studies was assessed using the Newcastle-Ottawa Scale (NOS) and Risk Of Bias In Non-randomized Studies of Interventions (ROBINS-I) criteria. Domains included selection of cohorts, comparability, and outcome assessment. Studies were classified as low, moderate, or high risk of bias.

All methodological tools and assessment instruments used in this review, including the PRISMA 2020 guidelines [10], the NOS [11], and the ROBINS-I tool [12], are publicly available and free for academic and non-commercial use. The Gastrografin challenge protocol and radiological predictors applied in the included studies are standard clinical practices and do not require specific licensing [13,14]. Relevant citations for these tools have been provided in the reference list and cited within the text and tables where applicable.

Data Synthesis

Given the substantial heterogeneity in study design, definitions of early surgery and NOM, and variability in outcome reporting, a meta-analysis was not performed. Results were synthesised narratively and organised according to prespecified primary outcomes (recurrence and major short-term complications) and secondary outcomes (mortality, length of stay, bowel resection and predictors of treatment failure).

Results

Study Selection

The electronic database search identified 1,342 records across MEDLINE, Embase, Scopus, and CENTRAL, with an additional 12 records obtained through manual reference screening, yielding a total of 1,354 records. After removal of 256 duplicates, 1,098 unique records were screened by title and abstract. Of these, 1,004 were excluded for irrelevance to aSBO or lack of comparative management data. Ninety-four full-text articles were assessed for eligibility. Eighty-five studies were excluded following full-text review due to non-adhesive or mixed aetiology SBO (n = 31), lack of comparison between operative management and NOM (n = 24), uncontrolled case series (n = 15), paediatric cohorts without isolated adhesive aetiology (n = 6), or non-original publications such as reviews or editorials (n = 9). Ultimately, nine studies met the inclusion criteria and were included in the qualitative synthesis. These comprised eight adult cohorts and one paediatric cohort. The study selection process is illustrated in the PRISMA flow diagram (Figure 1).

Overview and Characteristics of Included Studies

The nine included studies consisted of heterogeneous observational designs, including retrospective single-centre cohorts, prospective multicentre cohorts, and large population-based administrative analyses. Eight studies focused on adult populations, while one study evaluated paediatric patients. Sample sizes varied widely, ranging from 201 to 661 patients in single-centre studies and up to 27,904 patients in the largest population-based cohort. All studies specifically addressed aSBO and excluded alternative causes such as hernia, malignancy, inflammatory bowel disease, or early postoperative obstruction (Table 1).

**Table 1:** Study identification and design. aSBO = adhesive small bowel obstruction; DASBO = Danish Audit of Small Bowel Obstruction; NOM = non-operative management.

First author (year)	Country/region	Setting	Study design	Study period (index episodes)	Population (adult/pediatric)	Total N with aSBO included
Meier (2014) [1]	Switzerland (Geneva)	Single tertiary university hospital	Retrospective cohort	2004–2007	Adults	221
Lorentzen (2017) [2]	Denmark (Copenhagen)	Single university hospital	Retrospective cohort (surgically treated aSBO only)	2004–2013	Adults	478
Behman (2019) [3]	Canada (Ontario)	Population-based, all acute care hospitals	Retrospective, propensity-matched administrative cohort	2005–2014	Adults 18–80 years	27,904 (first aSBO episode)
Fung (2019) [4]	Canada (Ontario)	Population-based, all acute care hospitals	Retrospective administrative cohort	2005–2014	Adults	3,563 (aSBO operated after a trial of NOM)
Bauer (2015) [5]	USA (New York)	Single tertiary centre (Mount Sinai)	Retrospective cohort	2001–2011	Adults	460 (complete aSBO)
Ng (2023) [6]	Australia (Perth) & Malaysia	Two secondary/tertiary hospitals	Retrospective cohort	2015–2018	Adults ≥16 years	252 (aSBO)
Maienza (2023) [7]	France (Paris)	Single academic centre	Retrospective cohort, protocol-based	2008–2021	Adults	661 (adhesive post-op SBO)
Mortensen (2023) [8]	Denmark (6 hospitals, Zealand)	Prospective multicentre cohort	Prospective cohort (DASBO sub-study)	2021 (4-month inclusion)	Adults ≥18 years	201 (aSBO)
Hyak (2019) [9]	USA (Texas Children’s)	Single tertiary children’s hospital	Retrospective cohort	2011–2015	Pediatrics (≤18 years)	202 patients/258 admissions

Eligibility Criteria and Definitions of Management Strategies

Across the included studies, definitions of aSBO and treatment strategies varied. aSBO was generally defined based on clinical presentation, imaging findings, prior abdominal surgery, and exclusion of alternative causes. "Early" or "operative" management was variably defined as surgery during the index admission or within a specified time window, whereas conservative management or NOM typically involved bowel rest, nasogastric decompression, intravenous fluids, and close clinical monitoring, with escalation to surgery upon clinical or radiological deterioration (Table 2).

**Table 2:** Eligibility criteria and key definitions. CT = computed tomography; ICD-10 = International Classification of Diseases, 10th Revision; IBD = inflammatory bowel disease; NG = nasogastric; NOM = non-operative management; NPO = nil per os; aSBO = adhesive small bowel obstruction.

First author (year)	Main inclusion criteria	Key exclusion criteria	Definition of aSBO	Definition of "early surgery"	Definition of conservative/NOM
Meier (2014) [1]	Adults admitted with acute SBO, ICD-10 K56.5; first hospitalisation during 2004–2007	Large-bowel obstruction, hernia, early post-op SBO (<1 month), IBD, radiation enteritis, carcinomatosis	SBO coded K56.5 with prior abdominal surgery; adhesions presumed	Index episode managed surgically during that admission (exploratory laparotomy/adhesiolysis)	IV fluids, NG tube, bowel rest; no laparotomy during index episode
Lorentzen (2017) [2]	Emergency surgery for SBO caused by adhesions (NOMESCO SBS codes; ICD-10 K56.5–K56.7)	SBO due to tumour or tumour-related adhesions; lost to follow-up	Intra-operative confirmation that adhesions caused obstruction	NA – all included patients underwent surgery	NA: no primary NOM arm; recurrent episodes may be NOM or surgery
Behman (2019) [3]	Adults 18–80 years with primary adhesive/unspecified SBO (ICD-10 K56.5/K56.6), first ever episode	Any codes for non-adhesive causes (hernia, malignancy, IBD, radiotherapy); prior SBO in 5-year look-back; age >80 years	Administrative definition using K56.5/K56.6 after excluding other causes and prior SBO	Any operative billing code for adhesiolysis/lysis at index admission	Admission with aSBO discharged without operative procedure
Fung (2019) [4]	Adult patients with aSBO who underwent surgery after a trial of NOM	Non-adhesive causes, prior aSBO in 5-year look-back; age >80; surgery on day 0–1; surgery >14 days	Same as Behman (ICD-10 K56.x with adhesive definition)	Not “early vs NOM”; analyses by post-admission day (PAD 2 to ≥10) for surgery timing	All patients in cohort had initial NOM; NG decompression + bowel rest; those failing NOM proceeded to surgery
Bauer (2015) [5]	Adults with complete aSBO and prior abdominopelvic surgery	No previous abdominopelvic surgery; surgery in last 6 weeks; obstruction from other causes (hernia, tumour, Crohn’s)	Clinical + imaging diagnosis of complete SBO; intra-operative confirmation in operated patients	Surgery within 24 hours of admission (“immediate” group)	At least 24 hours of NG decompression, IV fluids, observation; surgery only if failure/deterioration
Ng (2023) [6]	≥16 years, SBO secondary to adhesions, CT documented	Virgin abdomen, immediate post-op, hernia, stricture, volvulus, IBD, malignancy, ileus; missing CT	CT criteria of SBO without alternative cause (aSBO)	Some patients had immediate surgery at clinician’s discretion; not a formal “early” threshold	NOM = NPO, NG tube, IV fluids ± water-soluble contrast (Gastrografin); decision to operate if clinical/contrast failure
Maienza (2023) [7]	Post-operative aSBO, CT confirmed, managed with Gastrografin protocol	Hernia, IBD, suspected carcinomatosis, pregnancy, Gastrografin allergy	Post-operative SBO in presence of prior abdominal surgery and CT evidence of adhesional obstruction	Group 1: emergency surgery at admission (peritonitis, hemodynamic instability, CT ischemia)	Group 2: standardised NOM with NG decompression + oral Gastrografin challenge; surgery if pain/vomiting during clamp or no contrast in colon at 8 h
Mortensen (2023) [8]	Adults with clinical/CT diagnosis of adhesional SBO in DASBO cohort	Non-adhesive causes (hernia, tumour, etc.)	Clinical + CT diagnosis with prior surgery/adhesions	Any surgery during index admission; 17 had delayed surgery after initial NOM	NOM = trial of non-operative treatment during index admission without surgery (bowel rest, NG, fluids), discharged without operation
Hyak (2019) [9]	Children ≤18 years with aSBO ≥4 weeks after abdominal surgery	Early post-op obstruction (<4 weeks); non-adhesive causes; complex genetic/metabolic syndromes	aSBO in previously operated children, coded SBO with prior surgery	“Urgent operation” at admission without NOM (usually high-grade obstruction or concern for ischemia)	NOM = NG decompression, fluids, correction of electrolytes; “failure of NOM” → delayed surgery

Baseline Patient Characteristics

Baseline demographic and clinical characteristics were broadly comparable between operative and non-operative groups in most studies, particularly with respect to age and sex. However, several studies demonstrated evidence of confounding by indication, with surgically managed patients often presenting with higher disease severity or worse clinical status. Larger administrative cohorts employed multivariable adjustment or propensity score matching to balance measured confounders between treatment groups. In paediatric populations, younger age, particularly infants under one year, was associated with a higher likelihood of surgical intervention (Table 3).

**Table 3:** Baseline patient characteristics by treatment group (high-level). ASA = American Society of Anesthesiologists score; CCI = Charlson Comorbidity Index; PAD = post-admission day; aSBO = adhesive small bowel obstruction.

First author (year)	Groups compared	N per group	Age (mean/median)	Sex (% female)	Prior abdominal surgery (%)	Prior SBO episodes (%)	Notes on comorbidity/severity
Meier (2014) [1]	Surgical vs conservative at index SBO	136 vs 85	Mean ~68 vs 65 years (similar)	~59% overall; similar by group	89% vs 94%	13.2% vs 22.4%	Surgical group had higher clinical severity score; 36% with score ≥3 vs 8% conservative
Behman (2019) [3]	Operative vs non-operative (propensity-matched)	6,186 vs 21,718 (before matching); 1:1 matched for analysis	Mean ~61 years adults	51.1% female overall	High prior surgery prevalence (via administrative data)	All first episode (0% prior SBO by design)	Matching balanced age, sex, comorbidity, income, rurality, hospital size
Bauer (2015) [5]	Immediate surgery (<24 h) vs initial NOM	106 vs 354	Adults; mean age similar between groups	Similar sex distribution	All had prior abdominopelvic surgery (by inclusion)	Some with previous SBO episodes (not highlighted as different)	Groups similar in age/comorbidity; all complete aSBO
Mortensen (2023) [8]	Operative vs non-operative index management	118 vs 83	Median ~73 vs 64 years	Slight female predominance	Majority with previous abdominal operations	Recurrent aSBO: 40/118 vs 17/83	NOM patients had poorer performance status; ASA and CCI similar
Maienza (2023) [7]	Immediate surgery (group 1) vs NOM (group 2)	148 vs 513	Median 66 vs 62 years	Both sexes; balanced	All with prior surgery (post-operative SBO)	Previous SBO more frequent in NOM group (p=0.002)	Higher lactate and more CT signs of ischemia in immediate-surgery group
Ng (2023) [6]	Operative (incl. failed NOM) vs successful NOM	90 vs 162	Mean 68.9 vs 68.1 years	Similar M:F ratio	Similar number of previous abdominal operations (1.9 vs 2.1)	Similar	Inflammatory markers and lactate similar between groups
Fung (2019) [4]	Operated after NOM, stratified by day of surgery (PAD 2–≥10)	3,563 total	Mean 69.1 years	64.8% female	Comorbidity burden higher in those with longer delay	All first aSBO episodes	Older, more comorbid and lower income more likely to have longer delay to surgery
Hyak (2019) [9]	Urgent operation vs failure of NOM vs successful NOM	31 vs 105 vs 122 cases	Median age 8 years overall	66% male overall	All had prior abdominal surgery	42% with prior aSBO	Children <1 year more likely to require surgery (OR 3.71)
Lorentzen (2017) [2]	Patients with vs without recurrent aSBO after surgery	58 vs 420	Median ~68 to 70 years	Recurrence group more often female (70.7% vs 61.2%)	87–90% prior abdominal surgery	10.3% vs 7.9% previous aSBO	Multiple/matted adhesions and fascial dehiscence more frequent in recurrence group

Management Approaches and Timing of Intervention

NOM strategies were largely consistent across studies and centred on bowel rest, nasogastric decompression, fluid resuscitation, and close monitoring. The integration of water-soluble contrast protocols in several cohorts represented an important evolution toward standardised escalation pathways. Surgical approaches predominantly involved open adhesiolysis, with selective use of laparoscopy. Timing of surgery varied by study design, allowing exploration of immediate versus delayed operative intervention and their respective outcomes (Table 4).

**Table 4:** Management details and timing. AXR = abdominal X-ray; PAD = post-admission day; NG = nasogastric; aSBO = adhesive small bowel obstruction.

First author (year)	Groups/strategies	Key timing definition	Components of NOM	Surgical approach	Bowel resection at index surgery
Meier (2014) [1]	Surgical vs conservative	Surgery during index admission vs no surgery	NG tube, IV fluids, bowel rest	Open laparotomy with adhesiolysis ± resection	44/136 (32.4%) required resection
Behman (2019) [3]	Operative vs non-operative at first aSBO	Surgery at first admission vs no surgery at first admission	Usual care (not detailed in admin data)	Mixed open/lap (billing codes)	Resection captured via billing but not core exposure
Fung (2019) [4]	Surgery after trial of NOM, stratified by PAD	PAD 2 to ≥10; all had at least one day of NOM	NG decompression, bowel rest; no patients operated PAD 0–1	Open vs laparoscopic (7.2% laparoscopic)	43.6% overall bowel resection; odds increased with delay
Bauer (2015) [5]	Immediate surgery vs initial NOM	Immediate = surgery <24 hours; Delayed = observed ≥24 hours, surgery if failure	NG tube, IV fluids, serial exams, imaging	Open laparotomy; some resections and stomas	Immediate: 29.2% resection; delayed: 28.0%; similar
Maienza (2023) [7]	Group 1: emergency surgery; Group 2: Gastrografin-assisted NOM ± delayed surgery	Surgery at admission vs surgery after Gastrografin trial vs success NOM	Standardised protocol: NG decompression 4 hours → 100 mL oral Gastrografin → clamp 8 hours → AXR; surgery if no colonic contrast or clinical deterioration	Mostly open; some laparoscopy at surgeon’s discretion	Immediate surgery group: 46% resection (for ischemia/necrosis); delayed surgery group: 10% resection (5 pts necrosis)
Mortensen (2023) [8]	Operative vs non-operative; subset with delayed surgery after NOM	Surgery during index admission; 17 had delayed op after trial of NOM	Bowel rest, NG, fluids, CT-guided decision	60 laparoscopy attempts (convert in 33, 55%); rest open	24/118 (21.7%) had resection; similar between open and (converted) lap groups
Ng (2023) [6]	Immediate surgery vs failed NOM vs successful NOM	Surgery at presentation vs surgery after NOM vs no surgery	NOM protocol inc. NG, IV fluids, Gastrografin with AXR at 6 h and repeat if needed	Standard open surgery for failures/immediate surgery	Resection status not primary endpoint (focus on predictors of NOM success)
Hyak (2019) [9]	Urgent operation vs failure of NOM vs successful NOM	Urgent op at admission; failures: surgery after ≥NOM hours	NOM = NG decompression, fluids, correction of electrolytes	Pediatric laparotomy/adhesiolysis	When excluding urgent ops, bowel resection 32.6% if surgery >48 hours vs 15.3% if ≤48 hours
Lorentzen (2017) [2]	Single operative cohort	Not focused on timing; all emergent aSBO surgery	NA	All open or lap-assisted adhesiolysis, coded by NOMESCO	28.1% resection overall

Short-Term Clinical Outcomes

Short-term outcomes demonstrated that, in appropriately selected adult patients, an initial trial of NOM did not confer excess mortality or major morbidity when compared with early surgery. However, a consistent signal emerged linking prolonged delays to surgery following failed conservative management with increased rates of bowel resection and serious postoperative complications. In paediatric cohorts, delayed operative intervention beyond 48 hours was similarly associated with substantially higher rates of bowel loss, underscoring the importance of timely escalation once NOM failure is evident (Table 5).

**Table 5:** Short-term (index admission/30-day) outcomes. HR = hazard ratio; LOS = length of stay; OR = odds ratio; aSBO = adhesive small bowel obstruction.

First author (year)	Comparison	Mortality (index/30-day)	Serious complications	Bowel ischemia/perforation	Index bowel resection	Length of stay (LOS)
Meier (2014) [1]	Surgical vs conservative	5-year mortality similar (age/sex-adjusted HR 1.1); early in-hospital mortality low in both	Not a main endpoint	Not specifically quantified by group	32.4% in surgical group	Surgical group had longer LOS than conservative
Bauer (2015) [5]	Immediate surgery vs delayed after NOM	No significant difference between groups	21% complications in both groups	Ischemia: 18.9% vs 15.7%; perforation: 7.5% vs 2.0% (higher in early group)	29.2% vs 28.0%	LOS similar; about one-quarter managed non-operatively and discharged
Fung (2019) [4]	PAD 2 vs later PAD for surgery	Overall 30-day mortality 7.3%; not significantly worse with longer delay after adjustment	14.8% serious complications; OR ↑ 7% per day delay	Not specifically separated	43.6% resection overall; OR ↑ 6% per day delay	Not primary reported outcome
Maienza (2023) [7]	Immediate surgery vs NOM ± delayed surgery	Mortality low; deaths mainly in very sick emergency-surgery group	Immediate surgery group had more severe presentations; conservative group avoided many complications	Intestinal ischemia in 33.1% immediate group vs ~0.9% late-ischemia resection after failed NOM	46% resection in immediate vs 10% in delayed surgery	Successful NOM median stay 3 days
Mortensen (2023) [8]	Operative vs non-operative	Higher 1-year all-cause mortality in operative group (HR 2.48) largely driven by early period	Peri-operative complications described but not fully separated in abstract	CT-suspected ischemia in 16.9% of operative group	21.7% resection; ~half laparoscopic attempt	Index LOS longer in operative group (median 6 vs 3 days)
Ng (2023) [6]	Operative vs successful NOM	30-day mortality not a primary focus; overall low	Complications not systematically reported	CT predictors (free fluid, transition point, absence of fecal sign) associated with failure of NOM, not directly with ischemia rates	Resection data not emphasised	LOS longer in operative/failure group vs successful NOM
Hyak (2019) [9]	Urgent op vs failure of NOM vs success NOM	Pediatric mortality rare	Complications not detailed in abstract; key outcome was resection	Bowel loss more common when op delayed >48 hours	Resection 32.6% if surgery after 48 hours vs 15.3% if ≤48 hours	LOS longest in failure-of-NOM group
Behman (2019) [3]	Operative vs non-operative (index)	Short-term mortality similar; study focused on recurrence	Peri-operative complications not granular in abstract	Not central	Some had bowel resection at index op	LOS not primary focus
Lorentzen (2017) [2]	Recurrent vs no recurrent aSBO after surgery	30-day outcomes reported but not key for recurrence analysis	Surgical complications incl. fascial dehiscence (risk factor for recurrence)	Iatrogenic bowel lesions recorded	28.1% resection	LOS not central

Long-Term Outcomes: Recurrence and Durability of Treatment

Long-term follow-up data from adult cohorts revealed a clear and consistent reduction in recurrent aSBO among patients managed operatively during the index admission. Across studies with extended follow-up, operative management reduced the risk of recurrence by approximately one-third to two-thirds compared with non-operative strategies. This recurrence benefit persisted across diverse study designs and healthcare systems, suggesting a durable protective effect of surgical adhesiolysis. Long-term recurrence data were not available for paediatric populations (Table 6).

**Table 6:** Long-term outcomes: recurrence and mortality. CI = confidence interval; HR = hazard ratio; NA = not applicable/not reported; aSBO = adhesive small bowel obstruction.

First author (year)	Main comparison	Follow-up duration	Recurrence of SBO – operative group	Recurrence of SBO – conservative/NOM group	Effect estimate for recurrence	Longer-term mortality
Meier (2014) [1]	Surgical vs conservative	Median 4.7 years	19/136 (14.0%) re-hospitalised for SBO	25/85 (29.4%) re-hospitalised	HR 0.5 (95% CI 0.3–0.9) favouring surgery	5-year mortality similar; age/sex-adjusted HR 1.1 (0.6–2.1)
Behman (2019) [3]	Operative vs non-operative first episode	Up to 5 years	13.0% recurrence	21.3% recurrence	HR 0.62 (0.56–0.68) favouring surgery	Mortality modelled as competing risk; no big disadvantage reported for surgery
Mortensen (2023) [8]	Operative vs non-operative	1 year	Recurrence-free survival 92.5%	66.60%	HR 0.22 (0.10–0.48) favouring surgery	1-y mortality higher with surgery: HR 2.48 (1.13–5.46)
Lorentzen (2017) [2]	Risk factors for recurrence after aSBO surgery	Median 2.2 years	58/478 (12.1%) recurrent aSBO (20.6% re-recurrence in those)	NA	Female sex, multiple/matted adhesions, fascial dehiscence ↑ recurrence; resection ↓ recurrence (HR 0.47)	Mortality assessed as competing risk; not main endpoint
Maienza (2023) [7]	Immediate surgery vs Gastrografin protocol	In-hospital + limited longer term	Recurrent SBO not a primary endpoint; protocol data mainly acute	–	–	Data mostly acute; long-term not central
Fung (2019) [4]	Length of NOM before surgery and outcomes	30-day outcomes	Not focused on recurrence	–	–	30-day mortality analysed vs delay; no effect of delay after adjustment
Ng (2023) [6]	Predictors of successful NOM	Short-term; recurrence not emphasis	–	–	–	–
Hyak (2019) [9]	Pediatric aSBO management	Short-term	–	–	–	–

Predictors of Non-operative Failure and Recurrence

Several studies explored predictors that may guide early decision-making. Radiologic features on CT imaging, including a clear transition point, free intraperitoneal fluid, and absence of the small bowel feces sign, were consistently associated with failure of NOM. Among surgically treated patients, anatomical factors such as multiple or matted adhesions and postoperative fascial dehiscence increased recurrence risk, whereas bowel resection during index surgery appeared protective. Delays to surgery after NOM failure emerged as a modifiable risk factor for adverse outcomes (Table 7).

**Table 7:** Predictors of NOM success/failure or recurrence (secondary aim). AXR = abdominal X-ray; HR = hazard ratio; NOM = non-operative management; OR = odds ratio; PAD = post-admission day; aSBO = adhesive small bowel obstruction.

First author (year)	Outcome modelled	Predictors associated with higher risk (NOM failure or recurrence)	Predictors associated with lower risk
Ng (2023) [6]	Need for surgery (failure of NOM) in aSBO	CT: definitive transition point (OR 2.67), free fluid (OR 2.11), absence of small bowel feces sign (OR 1.70)	Presence of contrast in colon after Gastrografin on AXR strongly predicted successful NOM (OR 3.83)
Lorentzen (2017) [2]	Recurrent aSBO after surgery	Female sex (HR 2.00), multiple/matted adhesions (HR 1.72), fascial dehiscence (HR 3.26)	Intestinal resection at index surgery decreased recurrence risk (HR 0.47)
Fung (2019) [4]	Serious complications and resection after surgery for aSBO following NOM	Each additional day before surgery ↑ odds of serious complications (OR 1.07/day) and resection (OR 1.06/day)	Earlier surgery after failed NOM (PAD 2–3) associated with lower complication and resection rates
Hyak (2019) [9]	Operation and bowel resection in children	Age <1 year (OR 3.71) ↑ need for operation; delayed surgery >48 hours ↑ resection risk (32.6% vs 15.3%)	Older children and those with prior aSBO episodes were more likely to succeed with NOM
Maienza (2023) [7]	Failure of Gastrografin-based NOM	Moderate/large peritoneal effusion more common in failures (group 2a vs 2b)	Previous SBO episode more frequent in successful NOM (37.2% vs 24.6%)

Risk of Bias and Overall Evidence Quality

Risk of bias assessment indicated an overall moderate quality of evidence. Population-based and prospective cohorts demonstrated lower risk of bias, particularly for recurrence outcomes, due to robust adjustment for confounding and comprehensive follow-up. In contrast, single-centre retrospective studies were more susceptible to confounding by indication and selection bias. These limitations primarily affect interpretation of short-term outcomes, while recurrence findings from larger cohorts appeared more reliable and consistent (Table 8).

**Table 8:** Risk of bias assessment (NOS style). aSBO = adhesive small bowel obstruction; ROBINS-I = Risk Of Bias In Non-randomized Studies of Interventions; NOS = Newcastle–Ottawa Scale.

First author (year)	Study design	Selection (representative cohort, clear exposure, outcome not present at start)	Comparability (control of confounders)	Outcome (assessment, follow-up)	Overall risk of bias
Meier (2014) [1]	Retrospective single-centre cohort	Clear inclusion/exclusion; first “index” SBO episode defined	Limited adjustment (mainly age/sex); potential residual confounding	Outcomes from hospital records + phone follow-up to 2011	Moderate
Lorentzen (2017) [2]	Retrospective cohort	Large, well-defined surgically treated aSBO cohort	Multivariable Cox for recurrence; adjusts for key factors	Complete follow-up via Danish registry; events well captured	Low–moderate
Behman (2019) [3]	Population-based administrative cohort	Very large, representative; first-episode aSBO; clear exposure definitions	Propensity-matched; extensive adjustment for patient & hospital factors	Longitudinal outcomes via linked admin data; competing risk models	Low (for recurrence question)
Fung (2019) [4]	Population-based administrative cohort (surgery after NOM)	Good capture of operated patients after NOM	Multivariable hierarchical logistic regression; adjusts for key confounders	30-day outcomes from validated admin data	Low–moderate
Bauer (2015) [5]	Retrospective single-centre cohort	Clear inclusion (complete aSBO) but surgeon decision influences group allocation	No formal adjustment for confounding; groups “similar” but potential bias	Outcomes from chart review; no long-term follow-up	Moderate–high
Ng (2023) [6]	Retrospective cohort	Consecutive cases with clear inclusion/exclusion	Multivariable logistic regression for predictors; some confounders adjusted	Short-term outcomes; CT read by blinded radiologist	Moderate
Maienza (2023) [7]	Retrospective single-centre with protocol	Consecutive protocol patients; clear groups	Mainly unadjusted comparisons; selection into emergency surgery vs NOM may be biased	Long inclusion period, but outcomes well documented; follow-up mainly in-hospital	Moderate–high
Mortensen (2023) [8]	Prospective multicentre cohort	Consecutive patients, clear definition; minimal selection bias	Multivariable Cox adjusted for age, comorbidity, treatment, etc.	One-year outcomes from unified electronic system; no loss to follow-up	Low
Hyak (2019) [9]	Retrospective pediatric cohort	Tertiary centre; well-defined aSBO cases	Some regression modelling, but selection of urgent vs NOM is clinician-driven	Outcomes from chart review; no registry linkage	Moderate

Discussion 

Principle Findings

This systematic study indicates that initial NOM is safe for hemodynamically stable patients with aSBO, whereas early operational care significantly decreases long-term recurrence. In multiple extensive, modern cohorts, early surgical intervention reduced the incidence of recurrence by roughly 40-60% in comparison with NOM [6-9]. Notwithstanding this advantage, long-term mortality exhibited no significant difference across techniques, suggesting that survival is influenced more by patient characteristics than by management decisions [7-9].

Interpretation of Short-Term Outcomes

Short-term morbidity, ischemia, and 30-day mortality were comparable between early surgery and a structured trial of NOM in stable patients [6,10,11-13]. The majority of partial aSBO cases resolve non-operatively, and conservative management for up to 72 hours is supported by prior systematic reviews and recommendations [14-17]. Several studies reported that excessive delays after NOM failure increase adverse outcomes: each additional day before surgery is associated with higher complication and resection rates [12,18,19]. Water-soluble contrast protocols, widely validated in both diagnostic and therapeutic roles, improved resolution rates and reduced hospital stay without increasing missed ischemia [13,20-22].

Predictors of NOM Failure 

Radiologic features, such as a definite transition point, free intraperitoneal fluid, closed-loop obstruction, and absence of the small-bowel feces sign, were strong predictors of failed conservative management [14,23,24]. These findings echo previously validated CT-based risk models for bowel ischemia and strangulation [25,26]. Incorporating these predictors into standardised care pathways may reduce unnecessary delays to operative intervention [27,28].

*Long-Term Outcomes and Recurrence* 

Long-term follow-up consistently favored early operative management across adult cohorts [7-9]. Prior population studies have shown similar trends, noting that repeated episodes treated with NOM accumulate future obstruction risk, whereas adhesiolysis at the index event offers durable recurrence reduction [29-32]. Minimally invasive adhesiolysis may further reduce long-term adhesion formation and subsequent SBO, although high-quality evidence remains limited [33,34]. The burden of recurrent aSBO contributes significantly to cumulative morbidity and healthcare utilisation [21,29,35].

Adults Versus Pediatrics Implications

Only one included study addressed paediatric aSBO, yet important distinctions emerged. NOM succeeded in over half of cases, but infants <1 year had substantially higher odds of requiring surgery, and delays >48 hours increased bowel resection rates [15]. These findings align with paediatric surgical reviews emphasizing earlier operative thresholds in young children due to limited physiological reserve and higher strangulation risk [19,35]. Due to scarce long-term paediatric data, extrapolation from adult outcomes should be done cautiously.

*Strengths and Limitations* 

Strengths of this review include comprehensive search methods, inclusion of adult and paediatric data, and evaluation of both short- and long-term outcomes. The main limitations arise from observational study designs, confounding by indication, and heterogeneity in definitions of early surgery, NOM duration, and outcome reporting. These limitations mirror challenges noted in earlier systematic reviews and guidelines [6,14,17]. The inability to perform meta-analysis reflects variability in reporting and outcome measurement across the literature. Paediatric evidence remains particularly limited [33-35].

Clinical Implications

For stable adults, a monitored 48- to 72-hour trial of NOM is supported by consistent evidence and guideline recommendations [17,22]. Early surgery should be prioritised when CT shows high-risk features or when symptoms fail to improve. Patients treated non-operatively must be counselled regarding recurrence risk, while those undergoing surgery should be informed about short-term morbidity and potential preventive benefits. For children, especially infants, earlier operative intervention may be indicated if no improvement occurs within 24-48 hours [15,19]. The potential benefits of laparoscopy and adhesion-prevention strategies, although promising, require further validation [33-35].

Future Directions

Future studies should develop and validate predictive models combining CT features, biomarkers, and clinical variables to guide individualised management. Prospective trials comparing standardised NOM protocols with early surgery are needed, as are multi-centre paediatric registries to address current knowledge gaps. Research into adhesion-prevention technologies, including minimally invasive surgery and barrier agents, may also reduce long-term recurrence burden [34-38].

## Conclusions

This systematic review demonstrates that both early surgery and a structured trial of NOM play important roles in the management of aSBO. In hemodynamically stable adult patients without signs of strangulation, initial NOM is safe, particularly when supported by water-soluble contrast protocols and close monitoring; however, early surgical intervention offers a clear and consistent reduction in long-term recurrence. Delayed surgery following failed conservative management is associated with increased morbidity and higher bowel resection rates, highlighting the importance of timely decision-making guided by clinical and radiological predictors.

In paediatric patients, especially infants, the safe observation window is narrower, and earlier surgical intervention may be warranted. Future research should focus on prospective comparative studies, predictive risk models, and paediatric-specific data to optimise patient selection and timing of intervention.
